# Association between triglyceride-glucose index and activities of daily living disability among middle-aged and older patients with arthritis: longitudinal evidence from CHARLS

**DOI:** 10.3389/fmed.2025.1618606

**Published:** 2025-07-09

**Authors:** Liang Ma, Yu-long Mu, Zhuo-ming Liu, Shu-wei Jiang, De-qiang Li

**Affiliations:** ^1^Department of Orthopaedics, Qilu Hospital of Shandong University, Jinan, Shandong, China; ^2^The First Clinical College of Shandong University, Jinan, Shandong, China; ^3^Weihai Maternal and Child Health Hospital, Weihai, Shandong, China

**Keywords:** triglyceride-glucose index, arthritis, activities of daily living, aging, CHARLS

## Abstract

**Objective:**

To investigate the longitudinal association between the triglyceride-glucose (TyG) index and activities of daily living (ADL) disability in middle-aged and older adults with arthritis.

**Methods:**

We analyzed data from the China Health and Retirement Longitudinal Study (CHARLS, 2015–2018), including 2,695 arthritis patients without baseline ADL disability. The TyG index was calculated as ln [fasting triglycerides (mg/dL) × fasting plasma glucose (mg/dL)/2]. ADL disability was defined as a score of ≥ 1 based on combined basic and instrumental ADL assessments. Multivariable Cox proportional hazards models were employed to analyze the association, with potential non-linear relationship explored using restricted cubic splines.

**Results:**

Over a median follow-up of 35.98 months, 369 participants (13.69%) developed ADL disability. In fully adjusted models, each 1-unit increase in TyG index was associated with a 26% elevated risk of ADL disability (Hazard ratio [HR] = 1.26, 95% confidence interval [CI]: 1.05–1.51). Compared to the Low TyG, the moderate-High TyG and High TyG showed 40% (HR = 1.40, 95%CI: 1.02–1.92) and 64% (HR = 1.64, 95%CI: 1.18–2.29) increased risks, respectively (*P*-trend = 0.003). Restricted cubic spline analysis revealed that higher levels of TyG index (> 8.65) were associated with progressively higher ADL disability risk. Subgroup analyses indicated greater risk amplification in younger patients (< 60 years: High TyG vs. Low TyG HR = 1.98, 95%CI: 1.09–3.60). Sensitivity analyses showed that these associations remained statistically significant across multiple analytic approaches, including analyses of unimputed data (HR = 1.43, 95% CI: 1.10–1.86), weighted Cox models, directed acyclic graph-based minimum adequate adjustment, and competing-risks models.

**Conclusion:**

Elevated TyG index shows a significant independent association with ADL disability in arthritis patients. These findings provide mechanistic support for the “metabolic-joint axis” hypothesis and suggest that metabolic monitoring might facilitate identification of individuals with elevated risk profiles of functional decline. The TyG index may serve as an economical risk assessment tool in primary care settings.

## 1 Introduction

Arthritis, a degenerative inflammatory disease strongly associated with aging, primarily manifests as two subtypes: osteoarthritis and rheumatoid arthritis. As a leading global cause of disability, its burden is escalating rapidly with population aging (1). The Health, Well-Being, and Aging in Latin America and the Caribbean (SABE) study revealed that 23.8%–55.6% of arthritis patients aged ≥ 60 years experience limitations in activities of daily living (ADL) (2), while Mexico’s The Community Oriented Program for the Control of Rheumatic Diseases (COPCORD) project further demonstrated a 21.4% arthritis-related disability rate among those over 65 (3). Projections from the US National Health Interview Survey indicate that ADL disability prevalence among arthritis patients could exceed 11.4% by 2040 (4). ADL disability not only causes a precipitous decline in patients’ quality of life, but also leads to an annual wage loss of more than $65 billion and direct healthcare costs of more than $100 billion (5, 6). While traditional views attribute ADL disability to cartilage degeneration and mechanical stress, emerging evidence suggests metabolic dysregulation may accelerate functional decline through the “metabolic-joint axis” (7, 8).

Type 2 diabetes mellitus is identified as an independent risk factor for arthritis, with pathogenic mechanisms involving hyperglycemia-induced oxidative stress, chronic inflammation (e.g., IL-6 and TNF-α upregulation), and insulin resistance (IR)-associated muscle atrophy and neuropathy (9, 10). Notably, IR itself—beyond being a prediabetic marker—directly impairs joint function by disrupting cartilage matrix synthesis (collagen-proteoglycan imbalance) and exacerbating systemic low-grade inflammation (11, 12).

The triglyceride-glucose (TyG) index, mathematically expressed as ln[fasting triglycerides (mg/dL) × fasting glucose (mg/dL)/2], integrates lipid-glucose co-regulation dynamics. This dual-marker approach captures both β-cell dysfunction (reflected by glucose elevation) and dysregulated adipose tissue lipolysis (manifested as triglyceride accumulation), thereby providing higher sensitivity for early insulin signaling impairment detection compared to isolated hyperglycemia or dyslipidemia (13). As a well-validated insulin resistance biomarker showing robust correlations with both hyperinsulinemic-euglycemic clamp measurements and HOMA-IR (14, 15), the TyG index offers exceptional clinical practicality in primary healthcare. Unlike the clamp technique (labor-intensive and costly) or HOMA-IR (requiring precise insulin assays), the TyG index requires only fasting lipid and glucose measurements (16). However, existing research predominantly focuses on cardiovascular outcomes, leaving its risk association for ADL disability in arthritis populations unexplored.

Utilizing the China Health and Retirement Longitudinal Study (CHARLS) cohort, this study examines the association between TyG index and ADL disability risk among middle-aged and older adults with arthritis. We hypothesize that the TyG index, as a composite metabolic indicator, is associated with increased risk of ADL disability. Demonstration of this association could inform more comprehensive intervention strategies, potentially integrating metabolic monitoring with joint care approaches. This aligns with WHO’s healthy aging objectives by identifying a modifiable biomarker for multifactorial chronic disease management.

## 2 Materials and methods

### 2.1 Data sources

This study utilized data from CHARLS database to conduct a three-year prospective cohort analysis (2015–2018). A three-year observation window was adopted to adequately capture longitudinal variations in the TyG index and ADL disability while mitigating follow-up attrition risks. CHARLS, a nationally representative cohort of community-dwelling adults aged ≥ 45 years across 28 Chinese provinces, initially enrolled 17,708 participants from 150 county-level districts during its baseline survey (2011). Subsequent follow-ups were conducted biennially or triennially through 2020. The study protocol received ethical approval from the Peking University Biomedical Ethics Committee (IRB00001052–11015), with written informed consent obtained from all participants.

### 2.2 Study population and outcome

**FIGURE 1:**
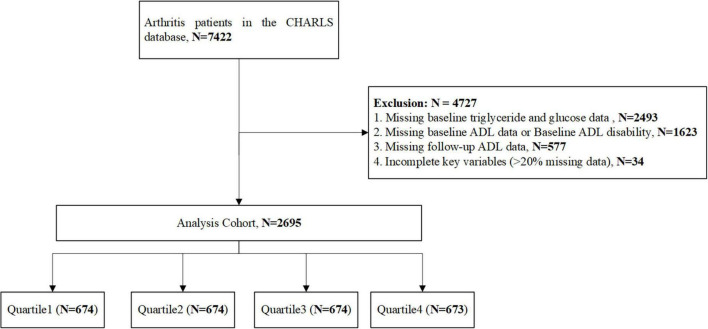
Flow chart of patients selection.

The study population comprised arthritis patients identified from the CHARLS database. Detailed inclusion flow is illustrated in Figure 1. The primary outcome was the occurrence of ADL disability.

### 2.3 Inclusion and exclusion criteria

Inclusion criteria:

(1) Patients diagnosed with arthritis in the 2015 wave: The diagnosis of arthritis was based on self-reported medical history data collected through standardized questionnaires. Participants who answered “yes” to the structured question “Have you ever been diagnosed with arthritis by a doctor?” during baseline and follow-up surveys were considered to have arthritis
(2) Complete fasting blood glucose and triglyceride data in the 2015 wave (fasting ≥ 8 h)
(3) Complete ADL assessment data at both baseline (2015) and follow-up (2018) waves

Exclusion criteria:

(1) pre-existing ADL disability in the 2015 wave
(2) > 20% missing data across variables

### 2.4 Data extraction

#### 2.4.1 Assessment of the TyG index

The following formula was used to determine the TyG index: ln [triglyceride concentration (mg/dL) × fasting blood glucose concentration (mg/dL)/2].

#### 2.4.2 ADL disability assessment

Activities of Daily Living (ADL) disability was assessed through two components: basic ADL (BADL, 6 items) and instrumental ADL (IADL, 5 items). BADL quantified essential self-care abilities: bathing, dressing, indoor mobility, toileting, feeding, and continence control. IADL evaluated complex task performance: housekeeping, cooking, shopping, financial management, and medication adherence. Existing evidence supports BADL and IADL as independent predictors of functional disability in older adults (17, 18). Each item was scored dichotomously (0 = independent, 1 = assistance required). The total ADL score (range 0–11) was derived from the sum of BADL (6 items) and IADL (5 items) scores, with higher values indicating greater disability. Using a validated cutoff, participants with total scores ≥ 1 were classified as having ADL disability (coded 1), while scores = 0 indicated functional independence (coded 0) (19).

#### 2.4.3 Covariates

The analysis incorporated covariates spanning four domains: sociodemographic characteristics, health behaviors and comorbidities, physical pain assessment, and muscle strength measurements. Sociodemographic covariates included age (continuous), sex (male/female), residence (urban/rural), highest educational attainment (illiterate, primary school, middle school, high school or above), and annual household income (continuous). Educational classification followed self-reported criteria: illiteracy was defined as no formal education; completion of elementary curriculum or demonstrated basic literacy skills qualified as primary education; middle school graduation denoted middle school level; high school or higher graduation indicated high school or above. Health behavior covariates comprised body mass index (BMI, continuous), smoking history (≥ 100 cumulative cigarettes smoked in lifetime), and alcohol consumption (abstinence in the past year). Diagnostic criteria for comorbidities were as follows: hypertension required either self-reported physician diagnosis or on-site measurement of systolic blood pressure ≥ 140 mmHg and/or diastolic blood pressure ≥ 90 mmHg; diabetes encompassed self-reported diagnosis or laboratory-confirmed fasting glucose abnormalities/impaired glucose tolerance. Physical pain was assessed using a standardized questionnaire capturing presence/absence of pain in 12 anatomical regions (head, shoulders, arms, wrists, fingers, back, waist, hips, legs, knees, ankles) coded as binary variables (yes/no). Grip strength was measured bilaterally using a Jamar Plus + digital dynamometer with two trials per hand. Participants unable to complete testing due to severe pain, joint swelling, or postoperative conditions were excluded. The maximum grip strength value from either hand was analyzed.

### 2.5 Statistical analysis

All statistical analyses were performed using R software (version 4.4.0). To ensure data quality, potential outliers were first identified and treated using the interquartile range (IQR) method, with all outliers treated as missing values. For missing data, variables with > 20% missingness were excluded, while those with ≤ 20% missingness were handled using multiple imputation by chained equations.

The study subjects were divided into 4 groups based on quartiles of TyG index levels, namely Low TyG, Moderate-Low TyG, Moderate-High TyG, and High TyG. Continuous variables were described as mean ± standard deviation for normally distributed data (compared using one-way ANOVA) or median (IQR) for non-normally distributed data (compared using Kruskal-Wallis test). Categorical variables were presented as counts (percentages) [n (%)], with between-group differences assessed by χ^2^ test or Fisher’s exact test. Additionally, participants were categorized into independence and disability groups based on ADL disability occurrence during follow-up.

To examine the association between TyG index and ADL disability in arthritis patients, Cox proportional hazards models were employed to estimate hazard ratios (HR) and 95% confidence intervals (CI). Three progressively adjusted models were constructed: Model 1 adjusted for age, sex (female/male), residence (urban/rural), education (illiteracy/elementary school/middle school/high school or above) and Income; Model 2 additionally adjusted for bmi, smoking (no/yes), drinking (no/yes), hypertension (no/yes) and diabetes (no/yes); Model 3 further adjusted for headache (no/yes), shoulder pain (no/yes), arm pain (no/yes), wrist pain (no/yes), finger pain (no/yes), back pain (no/yes), lumbar pain (no/yes), hip pain (no/yes), leg pain (no/yes), knee pain (no/yes), ankle pain (no/yes) and grip strength. TyG index was analyzed both as a continuous variable (per SD increment) and categorical variable (quartiles).

To prevent overfitting, variance inflation factors (VIF) were calculated (VIF ≥ 5 indicating multicollinearity). Potential non-linear relationship were explored using restricted cubic splines (RCS, 4 knots). Subgroup analyses were performed by stratifying participants according to age (< 60 years/ > = 60 years), sex (female/male), residence (urban/rural), education (illiteracy/elementary school/middle school/high school or above), BMI (< 25 kg/m^2^/ > = 25 kg/m^2^), smoking (no/yes), drinking (no/yes), hypertension (no/yes) and diabetes (no/yes). To ensure the robustness of our findings, we conducted comprehensive sensitivity analyses employing multiple advanced statistical approaches. First, a robust weighted Cox proportional hazards model was utilized, with weights derived from deviance residuals, to minimize the potential influence of outliers on effect estimates. Second, we performed complete-case analyses using unimputed raw data (Outliers were treated as missing values) to evaluate potential bias introduced by missing data. Third, covariates were systematically adjusted based on the minimal sufficient adjustment set (MSAS) derived from causal directed acyclic graphs (DAGs) to strengthen causal inference (Supplementary Figure 1). The DAGs-based MSAS adjustment included age, sex (female/male), education (illiteracy/elementary school/middle school/high school or above), income, BMI, diabetes (no/yes), finger pain (no/yes), and knee pain (no/yes). Finally, to account for competing risks, we applied the Fine-Gray subdistribution hazards model when analyzing the risk of ADL disability, with death treated as a competing event. All statistical tests were two-tailed, with *P* < 0.05 considered statistically significant.

## 3 Results

### 3.1 Baseline characteristics of patients

This study ultimately included 2,695 eligible arthritis patients. As shown in Table 1, participants were stratified into four groups by TyG index quartiles: Low TyG (*n* = 674), Moderate-Low TyG (*n* = 674), Moderate-High TyG (*n* = 674), and High TyG (*n* = 673). Baseline characteristics revealed a cohort comprising 949 males (35.21%) and 1,746 females (64.79%), with a median age of 62 years. During a mean follow-up of 35.98 months, 369 patients (13.69%) developed ADL disability. Patients stratified by TyG quartiles exhibited significant intergroup differences in age, sex distribution, residential status, household income, BMI, smoking rate, hypertension prevalence, diabetes prevalence and upper limb pain incidence (all *P* < 0.05), while no significant differences were observed in other measured covariates. Notably, ADL disability rates showed a significant ascending trend across ascending TyG index quartiles (Low TyG:10.68% vs Moderate-Low TyG:13.65% vs Moderate-High TyG:14.39% vs High TyG:16.05%, *P* < 0.05).

**TABLE 1:** Baseline characteristics stratified by TyG index quartile groups.

Variable	Overall (*N* = 2695)	Low TyG (*N* = 674)	Moderate-Low TyG (*N* = 674)	Moderate-High TyG (*N* = 674)	High TyG (*N* = 673)	*P*
Age, years, median (IQR)	62.00 (56.00,68.00)	62.50 (57.00,68.00)	63.00 (56.00,69.00)	62.00 (56.00,68.00)	62.00 (55.00,67.00)	0.031
Sex, *n* (%)						<0.001
Female	1,746.00 (64.79)	373.00 (55.34)	445.00 (66.02)	451.00 (66.91)	477.00 (70.88)	
Male	949.00 (35.21)	301.00 (44.66)	229.00 (33.98)	223.00 (33.09)	196.00 (29.12)	
Residence, *n* (%)						<0.001
Urban	853.00 (31.65)	163.00 (24.18)	195.00 (28.93)	212.00 (31.45)	283.00 (42.05)	
Rural	1,842.00 (68.35)	511.00 (75.82)	479.00 (71.07)	462.00 (68.55)	390.00 (57.95)	
Education, *n* (%)						0.050
Illiteracy	1,450.00 (53.80)	384.00 (56.97)	387.00 (57.42)	343.00 (50.89)	336.00 (49.93)	
Elementary school	619.00 (22.97)	150.00 (22.26)	140.00 (20.77)	173.00 (25.67)	156.00 (23.18)	
Middle school	452.00 (16.77)	104.00 (15.43)	109.00 (16.17)	112.00 (16.62)	127.00 (18.87)	
High school or above	174.00 (6.46)	36.00 (5.34)	38.00 (5.64)	46.00 (6.82)	54.00 (8.02)	
Income, Ten thousand yuan, median (IQR)	0.28 (0.10,1.94)	0.23 (0.08,0.99)	0.30 (0.10,1.60)	0.29 (0.12,1.96)	0.47 (0.12,2.76)	<0.001
BMI, kg/m^2^, median (IQR)	23.76 (21.48,26.54)	22.12 (19.95,24.32)	23.27 (21.12,25.63)	24.54 (22.15,26.95)	25.64 (23.34,28.24)	<0.001
Smoking, *n* (%)						<0.001
No	2,102.00 (78.00)	479.00 (71.07)	522.00 (77.45)	549.00 (81.45)	552.00 (82.02)	
Yes	593.00 (22.00)	195.00 (28.93)	152.00 (22.55)	125.00 (18.55)	121.00 (17.98)	
Drinking, *n* (%)						0.065
No	1,931.00 (71.65)	456.00 (67.66)	488.00 (72.40)	493.00 (73.15)	494.00 (73.40)	
Yes	764.00 (28.35)	218.00 (32.34)	186.00 (27.60)	181.00 (26.85)	179.00 (26.60)	
Hypertension, *n* (%)						<0.001
No	1,201.00 (44.56)	381.00 (56.53)	311.00 (46.14)	271.00 (40.21)	238.00 (35.36)	
Yes	1,494.00 (55.44)	293.00 (43.47)	363.00 (53.86)	403.00 (59.79)	435.00 (64.64)	
Diabetes, *n* (%)						<0.001
No	2,126.00 (78.89)	616.00 (91.39)	579.00 (85.91)	525.00 (77.89)	406.00 (60.33)	
Yes	569.00 (21.11)	58.00 (8.61)	95.00 (14.09)	149.00 (22.11)	267.00 (39.67)	
Headache, *n* (%)						0.190
No	1,959.00 (72.69)	497.00 (73.74)	502.00 (74.48)	469.00 (69.58)	491.00 (72.96)	
Yes	736.00 (27.31)	177.00 (26.26)	172.00 (25.52)	205.00 (30.42)	182.00 (27.04)	
Shoulder pain, *n* (%)						0.135
No	1,925.00 (71.43)	476.00 (70.62)	503.00 (74.63)	465.00 (68.99)	481.00 (71.47)	
Yes	770.00 (28.57)	198.00 (29.38)	171.00 (25.37)	209.00 (31.01)	192.00 (28.53)	
Arm pain, *n* (%)						0.048
No	2,081.00 (77.22)	512.00 (75.96)	531.00 (78.78)	500.00 (74.18)	538.00 (79.94)	
Yes	614.00 (22.78)	162.00 (24.04)	143.00 (21.22)	174.00 (25.82)	135.00 (20.06)	
Wrist pain, *n* (%)						0.335
No	2,218.00 (82.30)	553.00 (82.05)	559.00 (82.94)	541.00 (80.27)	565.00 (83.95)	
Yes	477.00 (17.70)	121.00 (17.95)	115.00 (17.06)	133.00 (19.73)	108.00 (16.05)	
Finger pain, *n* (%)						0.500
No	2,186.00 (81.11)	546.00 (81.01)	545.00 (80.86)	537.00 (79.67)	558.00 (82.91)	
Yes	509.00 (18.89)	128.00 (18.99)	129.00 (19.14)	137.00 (20.33)	115.00 (17.09)	
Back pain, *n* (%)						0.108
No	2,082.00 (77.25)	502.00 (74.48)	532.00 (78.93)	514.00 (76.26)	534.00 (79.35)	
Yes	613.00 (22.75)	172.00 (25.52)	142.00 (21.07)	160.00 (23.74)	139.00 (20.65)	
Lumbar pain, *n* (%)						0.289
No	1,676.00 (62.19)	405.00 (60.09)	411.00 (60.98)	424.00 (62.91)	436.00 (64.78)	
Yes	1,019.00 (37.81)	269.00 (39.91)	263.00 (39.02)	250.00 (37.09)	237.00 (35.22)	
Hip pain, *n* (%)						0.065
No	2,349.00 (87.16)	580.00 (86.05)	594.00 (88.13)	573.00 (85.01)	602.00 (89.45)	
Yes	346.00 (12.84)	94.00 (13.95)	80.00 (11.87)	101.00 (14.99)	71.00 (10.55)	
Leg pain, *n* (%)						0.980
No	1,852.00 (68.72)	463.00 (68.69)	467.00 (69.29)	463.00 (68.69)	459.00 (68.20)	
Yes	843.00 (31.28)	211.00 (31.31)	207.00 (30.71)	211.00 (31.31)	214.00 (31.80)	
Knee pain, *n* (%)						0.580
No	1,777.00 (65.94)	445.00 (66.02)	455.00 (67.51)	431.00 (63.95)	446.00 (66.27)	
Yes	918.00 (34.06)	229.00 (33.98)	219.00 (32.49)	243.00 (36.05)	227.00 (33.73)	
Ankle pain, *n* (%)						0.113
No	2,236.00 (82.97)	550.00 (81.60)	565.00 (83.83)	546.00 (81.01)	575.00 (85.44)	
Yes	459.00 (17.03)	124.00 (18.40)	109.00 (16.17)	128.00 (18.99)	98.00 (14.56)	
Grip strength, kg, median (IQR)	27.80 (22.20,34.00)	28.10 (23.00,34.00)	27.20 (22.00,33.50)	27.50 (22.00,34.50)	27.10 (22.50,34.00)	0.183
ADL *disability*, *n* (%)						0.035
No	2,326.00 (86.31)	602.00 (89.32)	582.00 (86.35)	577.00 (85.61)	565.00 (83.95)	
Yes	369.00 (13.69)	72.00 (10.68)	92.00 (13.65)	97.00 (14.39)	108.00 (16.05)	

Low TyG: 7.29 < TyG ≤ 8.28, Moderate-Low TyG: 8.28 < TyG ≤ 8.65, Moderate-High TyG: 8.65 < TyG ≤ 9.11, High TyG: 9.11 < TyG ≤ 11.23. TyG, triglyceride-glucose index; ADL, activities of daily living; BMI, body mass index; IQR, interquartile range.

Supplementary Table 1 presents the comparative results stratified by ADL disability status (independent group, *n* = 2,326 vs disability group, *n* = 369). Significant between-group differences (*P* < 0.05) were observed for the following variables: age, residence, education level, household income, alcohol consumption, hypertension, diabetes, lumbar pain, hip pain, leg pain, knee pain, ankle pain, grip strength, and TyG index. No statistically significant differences were found for the remaining observed indicators.

### 3.2 TyG index and ADL disability in arthritis patients

**FIGURE 2:**
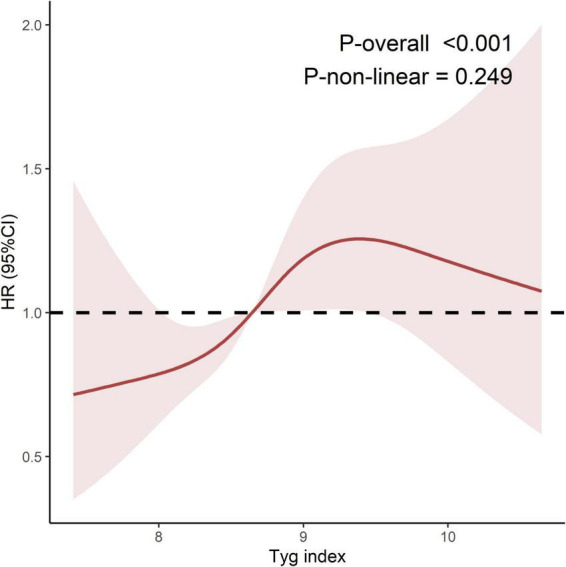
Restricted cubic spline regression of TyG index and ADL disability. TyG, triglyceride-glucose index; ADL, activities of daily living; CI, confidence interval; HR, Hazard ratio; Ref, Reference. Model adjusted for age, sex (female/male), Residence (urban/rural), education (illiteracy/elementary school/middle school/high school or above), Income, bmi, smoking (no/yes), drinking (no/yes), hypertension (no/yes), diabetes (no/yes, headache (no/yes), shoulder pain (no/yes), arm pain (no/yes), wrist pain (no/yes), finger pain (no/yes), back pain (no/yes), lumbar pain (no/yes), hip pain (no/yes), leg pain (no/yes), knee pain (no/yes), ankle pain (no/yes) and grip strength. The number of nodes was 4, and the reference value was 8.65.

Multivariable-adjusted Cox proportional hazards regression analysis demonstrated that TyG index maintained significant risk association for ADL disability risk after adjusting for confounders, whether analyzed as a continuous or categorical variable (Table 2). Specifically, in the fully adjusted model (Model 3), each 1-unit increase in TyG index was associated with a 26% elevated risk of ADL disability (HR = 1.26, 95% CI: 1.05–1.51). When stratified by TyG index quartiles, compared with Low TyG (reference), Moderate-High TyG and High TyG groups exhibited 40% (HR = 1.40, 95% CI: 1.02–1.92) and 64% (HR = 1.64, 95% CI: 1.18–2.29) increased risks, respectively, with a statistically significant monotonic trend across quartiles (*P*-trend = 0.003). RCS analysis showed that higher levels of TyG index (> 8.65) were associated with an increased risk of ADL disability (Figure 2).

**TABLE 2:** Association between TyG index and ADL disability.

	Continuous variable	TyG index (HR, 95% CI)				*P* for trend^a^
		Low TyG	Moderate-Low TyG	Moderate-High TyG	High TyG	
Model 1 HR (95%CI)	1.33 (1.13, 1.56)**	Ref.	1.30 (0.95, 1.77)	1.47 (1.08, 2.00)*	1.77 (1.31, 2.41)***	< 0.001
Model 2 HR (95%CI)	1.23 (1.03, 1.47)*	Ref.	1.26 (0.92, 1.72)	1.37 (1.00, 1.88)*	1.59 (1.14, 2.21)*	0.006
Model 3 HR (95%CI)	1.26 (1.05, 1.51)*	Ref.	1.25 (0.91, 1.71)	1.40 (1.02, 1.92)*	1.64 (1.18, 2.29)**	0.003

Low TyG: 7.29 < TyG ≤ 8.28, Moderate-Low TyG: 8.28 < TyG ≤ 8.65, Moderate-High TyG: 8.65 < TyG ≤ 9.11, High TyG: 9.11 < TyG ≤ 11.23. TyG, triglyceride-glucose index; ADL, activities of daily living; CI, confidence interval; HR, hazard ratio; Ref, reference. Model 1 was adjusted for age, sex (female/male), Residence (urban/rural), education (illiteracy/elementary school/middle school/high school or above) and Income. Model 2 was additionally adjusted for bmi, smoking (no/yes), drinking (no/yes), hypertension (no/yes) and diabetes (no/yes). Model 3 was additionally adjusted for headache (no/yes),shoulder pain (no/yes), arm pain (no/yes), wrist pain (no/yes), finger pain (no/yes), back pain (no/yes), lumbar pain (no/yes), hip pain (no/yes), leg pain (no/yes), knee pain (no/yes), ankle pain (no/yes) and grip strength.

*^a^P* for trend was estimated by including the quartile of the TyG *index* as a continuous variable. *P < 0.05; **P < 0.01; ***P < 0.001.

### 3.3 Subgroup analysis

Subgroup analyses stratified by demographic and clinical characteristics (age, sex, residence, education level, BMI, smoking status, alcohol consumption, hypertension, and diabetes status) were conducted to evaluate the robustness of TyG index-ADL disability association (Table 3). In the continuous variable model, each unit increment in TyG index demonstrated significantly elevated risks of ADL disability across multiple subgroups: participants aged < 60 years (HR = 1.46, 95% CI: 1.06–2.01), males (HR = 1.54, 95% CI: 1.13–2.11), rural residents (HR = 1.29, 95% CI: 1.03–1.60), those with elementary education (HR = 1.51, 95% CI: 1.01–2.26), individuals with BMI ≥ 25 kg/m^2^ (HR = 1.35, 95% CI: 1.02–1.79), current smokers (HR = 1.65, 95% CI: 1.13–2.42), non-drinkers (HR = 1.23, 95% CI: 1.00–1.52), and participants without hypertension (HR = 1.56, 95% CI: 1.14–2.13) or diabetes (HR = 1.26, 95% CI: 1.01–1.57). Notably, with ADL disability risk exhibiting monotonic elevation across ascending TyG index quartiles (*P*-trend < 0.001). Interaction analyses identified significant effect modification by age strata (*P*-interaction = 0.039), with particularly pronounced TyG-ADL disability association in the younger subgroup (< 60 years: High TyG vs Low TyG HR = 1.98, 95% CI: 1.09–3.60) compared to older counterparts. No other stratified variables demonstrated statistically significant interaction effects.

**TABLE 3:** Subgroup analyses of the association between TyG index and ADL disability.

Subgroup	Continuous variable	TyG index (HR, 95% CI)				*P* for trend^a^	*P* for interaction^b^
		Low TyG	Moderate-Low TyG	Moderate-High TyG	High TyG		
Age							0.039
< 60 years	1.46 (1.06, 2.01)*	Ref.	0.79 (0.40, 1.58)	1.87 (1.03, 3.41)*	1.98 (1.09, 3.60)*	0.003	
≥ 60 years	1.12 (0.89, 1.41)	Ref.	1.39 (0.97, 1.98)	1.24 (0.85, 1.81)	1.40 (0.93, 2.10)	0.191	
Sex							0.145
Female	1.12 (0.89, 1.41)	Ref.	1.03 (0.70, 1.53)	1.04 (0.70, 1.55)	1.25 (0.83, 1.88)	0.278	
Male	1.54 (1.13, 2.11)*	Ref.	1.52 (0.89, 2.57)	2.34 (1.38, 3.97)**	2.43 (1.36, 4.32)**	0.001	
Residence							0.476
Urban	1.26 (0.89, 1.79)	Ref.	0.76 (0.39, 1.50)	0.80 (0.40, 1.57)	1.15 (0.62, 2.13)	0.482	
Rural	1.29 (1.03, 1.60)*	Ref.	1.48 (1.03, 2.11)*	1.66 (1.15, 2.41)**	1.89 (1.27, 2.82)**	0.002	
Education							0.177
Illiteracy	1.18 (0.93, 1.48)	Ref.	1.36 (0.93, 2.00)	1.37 (0.92, 2.04)	1.49 (0.98, 2.27)	0.084	
Elementary school	1.51 (1.01, 2.26)*	Ref.	1.58 (0.76, 3.28)	1.99 (0.98, 4.02)	2.61 (1.22, 5.56)*	0.011	
Middle school	1.25 (0.68, 2.28)	Ref.	0.65 (0.23, 1.85)	0.70 (0.25, 1.99)	1.31 (0.48, 3.57)	0.523	
High school or above	2.72 (0.55, 13.49)	Ref.	0.03 (0.00, 1.04)	0.50 (0.03, 7.19)	0.46 (0.02, 9.21)	0.633	
BMI							0.909
< 25 kg/m^2^	1.19 (0.93, 1.53)	Ref.	1.34 (0.93, 1.93)	1.27 (0.85, 1.91)	1.78 (1.16, 2.73)**	0.016	
≥ 25 kg/m^2^	1.35 (1.02, 1.79)*	Ref.	0.99 (0.54, 1.82)	1.37 (0.77, 2.41)	1.42 (0.80, 2.49)	0.104	
Smoking							0.651
No	1.17 (0.95, 1.45)	Ref.	1.21 (0.83, 1.75)	1.25 (0.86, 1.82)	1.43 (0.97, 2.11)	0.080	
Yes	1.65 (1.13, 2.42)**	Ref.	1.28 (0.71, 2.32)	2.05 (1.10, 3.79)*	2.60 (1.32, 5.14)**	0.003	
Drinking							0.921
No	1.23 (1.00, 1.52)*	Ref.	1.24 (0.87, 1.78)	1.33 (0.92, 1.91)	1.49 (1.02, 2.19)*	0.045	
Yes	1.37 (0.93, 2.04)	Ref.	1.22 (0.64, 2.35)	1.77 (0.90, 3.50)	2.29 (1.14, 4.61)*	0.013	
Hypertension							0.589
No	1.56 (1.14, 2.13)*	Ref.	1.17 (0.71, 1.91)	1.39 (0.83, 2.34)	2.01 (1.19, 3.39)**	0.009	
Yes	1.10 (0.88, 1.39)	Ref.	1.32 (0.87, 2.01)	1.39 (0.92, 2.11)	1.43 (0.92, 2.23)	0.135	
Diabetes							0.270
No	1.26 (1.01, 1.57)*	Ref.	1.12 (0.80, 1.56)	1.37 (0.97, 1.94)	1.56 (1.07, 2.26)*	0.010	
Yes	1.25 (0.88, 1.76)	Ref.	2.76 (0.99, 7.68)	1.57 (0.58, 4.29)	2.33 (0.87, 6.25)	0.329	

Low TyG: 7.29 < TyG ≤ 8.28, Moderate-Low TyG: 8.28 < TyG ≤ 8.65, Moderate-High TyG: 8.65 < TyG ≤ 9.11, High TyG: 9.11 < TyG ≤ 11.23. TyG, triglyceride-glucose index; ADL, activities of daily living; CI, confidence interval; HR, hazard ratio; Ref, reference. Model adjusted for all variables except the subgroup variable, including age, sex (female/male), Residence (urban/rural), education (illiteracy/elementary school/middle school/high school or above), Income, bmi, smoking (no/yes), drinking (no/yes), hypertension (no/yes), diabetes (no/yes, headache (no/yes), shoulder pain (no/yes), arm pain (no/yes), wrist pain (no/yes), finger pain (no/yes), back pain (no/yes), lumbar pain (no/yes), hip pain (no/yes), leg pain (no/yes), knee pain (no/yes), ankle pain (no/yes) and grip strength. *^a^P* for trend was estimated by including the quartile of the TyG index as a continuous variable. *^b^P* for interaction was calculated by adding an interaction term of the quartile of the TyG index and the stratification variable in cox regression models. *P < 0.05; **P < 0.01; ***P < 0.001.

### 3.4 Sensitivity analysis

The sensitivity analyses consistently demonstrated the stability of our primary findings across different analytical approaches (Supplementary Table 2). In analyses using non-imputed data, each unit increase in TyG index remained significantly associated with ADL disability risk (HR = 1.43, 95% CI: 1.10–1.86). When examining TyG index by quartiles, we observed a consistent pattern of increasing risk estimates across successively higher quartiles (*P*-trend < 0.01). Specifically, participants in the High TyG quartile exhibited a 2.10-fold increased risk (HR = 2.10, 95% CI: 1.29–3.42) compared to the reference group, while those in the Moderate-High quartile showed a 1.74-fold increased risk (HR = 1.74, 95% CI: 1.09–2.78). These associations maintained statistical significance across various model specifications, including weighted Cox regression, DAG-based minimal sufficient adjustment, and competing risk analysis, supporting the robustness of our findings to different methodological assumptions.

## 4 Discussion

This study, which utilized the CHARLS longitudinal cohort, is the first to demonstrate a significant association between the TyG index and ADL disability in middle-aged and older adults with arthritis. Over a mean follow-up of 3 years, patients with elevated TyG indices exhibited a higher risk of ADL disability. Compared to the Low TyG, the High TyG was associated with a 64% increased risk of ADL disability, while each 1-unit increase in the TyG index corresponded to a 26% elevation in risk. These findings not only support the “metabolic-joint axis” theory but also suggest IR may contribute to functional decline in arthritis, offering novel therapeutic targets for clinical intervention.

Insulin resistance, a cardinal pathological feature of metabolic dysregulation, is characterized by progressive impairment of insulin-mediated glucose uptake and utilization in peripheral tissues, often occurring under normal or elevated circulating insulin levels. While the hyperinsulinemic-euglycemic clamp remains the gold standard for IR assessment, its clinical application is hindered by procedural complexity, time requirements, and high costs (20). Alternative biomarkers such as QUICKI and HOMA-IR, though widely used, face limitations due to their reliance on complex calculations or insulin assays (21, 22). In contrast, the TyG index has emerged as a practical and cost-effective alternative. Multiple studies confirm its strong correlation with clamp-measured insulin sensitivity (23, 24) and its superior diagnostic accuracy for metabolic syndrome compared to HOMA-IR, as evidenced by a significantly larger area under the ROC curve (15). Notably, the TyG index requires only routine fasting lipid and glucose measurements, enabling “minimal additional cost” implementation in primary care settings and large-scale epidemiological studies (25).

Prior research has predominantly focused on the relationship between metabolic syndrome and structural progression of arthritis. This study innovatively extends the investigation to functional outcomes. Consistent with the “metabolic arthropathy” hypothesis proposed by Zhuo et al. (7), elevated TyG indices may exacerbate functional impairment through multiple mechanisms: IR-induced hyperglycemia promotes “glucotoxicity,” leading to oxidative stress, accumulation of advanced glycation end products (AGEs), and inflammatory cytokine release, directly damaging joint tissues (26). Simultaneously, IR suppresses chondrocyte autophagy (e.g., reduced autophagic flux, enhanced Akt phosphorylation, and rpS6 protein synthesis), impairing metabolic waste clearance and accelerating cartilage degradation (27). In synovial cells, IR diminishes insulin’s inhibitory effects on matrix-degrading enzymes (MMP1 and MMP13) while activating the PI3K/mTOR/Akt/NF-κB pathway, fostering a pro-inflammatory microenvironment and sustained autophagy suppression (28). Additionally, IR-associated glucose metabolism dysregulation drives abnormal synovial glycolysis (e.g., upregulated phosphoglycerate kinase activity) and IL-34-mediated glycolytic amplification, promoting inflammatory macrophage polarization, Th1/Th17 cell activation, and synovial hyperplasia, thereby exacerbating joint destruction (29). IR further downregulates glucose transporters (Glut1/Glut4), reducing energy supply to cartilage and hastening degeneration (30). Collectively, these mechanisms disrupt joint metabolic homeostasis, establishing IR as a central driver of arthritis progression. The observed stronger association between TyG index and ADL disability in middle-aged participants (45–60 years) compared to their older counterparts (≥ 60 years) may reflect critical transitions in metabolic-joint pathophysiology during aging. In middle-aged individuals, several converging factors likely contribute to heightened metabolic vulnerability: (1) This life stage represents a pivotal window when early metabolic dysfunction begins to manifest clinically, yet age-related compensatory mechanisms remain underdeveloped. The musculoskeletal system retains sufficient functional reserve to reveal metabolic influences before being overwhelmed by advanced degenerative changes that dominate in later years. (2) Compared to older adults, middle-aged individuals typically maintain higher physical activity levels and greater mechanical loading demands, potentially amplifying the clinical impact of metabolic impairments on functional capacity.

Our findings support the integration of TyG index assessment into routine arthritis care to guide personalized treatment strategies. For patients with TyG levels > 8.65—particularly middle-aged individuals (45–60 years) who showed the strongest metabolic vulnerability—a dual approach targeting both metabolic dysfunction and joint health should be implemented. Clinicians should prioritize structured lifestyle interventions, including Mediterranean-style, low-glycemic diets and progressive resistance training, while considering adjunctive pharmacotherapy with insulin-sensitizing agents like metformin or GLP-1 receptor agonists for high-risk patients. Rehabilitation programs should incorporate metabolic monitoring alongside targeted periarticular muscle strengthening to disrupt the cycle of metabolic deterioration and functional decline. Nursing teams play a crucial role in patient education, monitoring adherence to lifestyle modifications, and facilitating coordination between physicians, dietitians, and physical therapists. At the healthcare system level, incorporating TyG screening into standard arthritis assessments would enable early identification of at-risk patients, allowing for timely intervention during the critical middle-age window when metabolic management may be most effective in preserving function.

Several limitations of this study warrant careful consideration when interpreting the findings. First, reliance on a single database may introduce regional and selection biases, potentially limiting the generalizability of our results. Second, the exclusive use of self-reported arthritis diagnoses represents a notable limitation, as participant recall or reporting inaccuracies may lead to disease misclassification. Importantly, such non-differential misclassification would typically bias effect estimates toward the null hypothesis, suggesting that our observed associations may represent conservative estimates of the true relationship between TyG index and ADL disability risk. While this limitation precludes definitive conclusions about arthritis-specific mechanisms, the consistent results across sensitivity analyses strengthen confidence in the overall metabolic risk association. Additionally, the inability to distinguish between osteoarthritis and rheumatoid arthritis subtypes represents a significant constraint, as these conditions may demonstrate distinct metabolic pathways and differential associations with functional decline. Finally, the single baseline measurement of TyG index precluded our ability to examine temporal metabolic changes in relation to disability progression. Future studies should incorporate clinically confirmed arthritis diagnoses with subtype differentiation in multicenter cohorts, while implementing serial TyG measurements to assess dynamic metabolic changes. Complementary mechanistic studies combining advanced joint imaging with metabolomic profiling could elucidate subtype-specific pathways linking metabolic dysregulation to functional decline. These approaches would provide more robust evidence for potential clinical applications of TyG index monitoring in arthritis management.

## 5 Conclusion

This study demonstrates a strong, independent association between elevated TyG index and increased risk of ADL disability in middle-aged and older adults with arthritis, showing progressively higher risks with increasing TyG levels. These findings suggest the potential value of incorporating metabolic profiling into joint care strategies, with the TyG index representing a practical screening measure for identifying high-risk individuals.

## Data Availability

The original contributions presented in this study are included in this article/Supplementary material, further inquiries can be directed to the corresponding author.
