# Supplementary material for: Association between triglyceride-glucose index and activities of daily living disability among middle-aged and older patients with arthritis: longitudinal evidence from CHARLS
Source: Front Med (Lausanne). 2025 Jul 9;12:1618606. doi: 10.3389/fmed.2025.1618606 (PMC12283620; doi:10.3389/fmed.2025.1618606)
Supplement: Supplementary file 1 [file Table_1.docx]

Supplementary Material

# Supplementary Tables

| Supplementary Table 1 Baseline Characteristics Stratified by ADL disability Groups | | | | |
| --- | --- | --- | --- | --- |
| Variable | Overall  (N =2695) | Independence  (N =2326) | Disability  (N = 369) | *P* |
| Age, years, median (IQR) | 62.00 (56.00,68.00) | 62.00 (56.00,68.00) | 65.00 (58.00,72.00) | <0.001 |
| Sex, n (%) |  |  |  | 0.294 |
| Female | 1,746.00 (64.79) | 1,498.00 (64.40) | 248.00 (67.21) | |
| Male | 949.00 (35.21) | 828.00 (35.60) | 121.00 (32.79) | |
| Residence, n (%) | |  |  | 0.003 |
| Urban | 853.00 (31.65) | 761.00 (32.72) | 92.00 (24.93) |  |
| Rural | 1,842.00 (68.35) | 1,565.00 (67.28) | 277.00 (75.07) | |
| Education, n (%) | |  |  | <0.001 |
| Elementary school or below | 1,450.00 (53.80) | 1,212.00 (52.11) | 238.00 (64.50) | |
| Elementary school | 619.00 (22.97) | 537.00 (23.09) | 82.00 (22.22) |  |
| Middle school | 452.00 (16.77) | 413.00 (17.76) | 39.00 (10.57) |  |
| High school or above | 174.00 (6.46) | 164.00 (7.05) | 10.00 (2.71) |  |
| Income,Ten thousand yuan, median (IQR) | 0.28 (0.10,1.94) | 0.29 (0.10,2.02) | 0.25 (0.10,1.29) | 0.031 |
| BMI, kg/m^2^, median (IQR) | 23.76 (21.48,26.54) | 23.76 (21.50,26.51) | 23.99 (21.37,26.83) | 0.954 |
| Smoking, n (%) | |  |  | 0.291 |
| No | 2,102.00 (78.00) | 1,822.00 (78.33) | 280.00 (75.88) | |
| Yes | 593.00 (22.00) | 504.00 (21.67) | 89.00 (24.12) |  |
| Drinking, n (%) | |  |  | 0.002 |
| No | 1,931.00 (71.65) | 1,642.00 (70.59) | 289.00 (78.32) | |
| Yes | 764.00 (28.35) | 684.00 (29.41) | 80.00 (21.68) |  |
| Hypertension, n (%) | |  |  | <0.001 |
| No | 1,201.00 (44.56) | 1,071.00 (46.04) | 130.00 (35.23) | |
| Yes | 1,494.00 (55.44) | 1,255.00 (53.96) | 239.00 (64.77) | |
| Diabetes, n (%) | |  |  | 0.013 |
| No | 2,126.00 (78.89) | 1,853.00 (79.66) | 273.00 (73.98) | |
| Yes | 569.00 (21.11) | 473.00 (20.34) | 96.00 (26.02) |  |
| Headache, n (%) | |  |  | 0.096 |
| No | 1,959.00 (72.69) | 1,704.00 (73.26) | 255.00 (69.11) | |
| Yes | 736.00 (27.31) | 622.00 (26.74) | 114.00 (30.89) | |
| Shoulder pain, n (%) | |  |  | 0.053 |
| No | 1,925.00 (71.43) | 1,677.00 (72.10) | 248.00 (67.21) | |
| Yes | 770.00 (28.57) | 649.00 (27.90) | 121.00 (32.79) | |
| Arm pain, n (%) | |  |  | 0.599 |
| No | 2,081.00 (77.22) | 1,800.00 (77.39) | 281.00 (76.15) | |
| Yes | 614.00 (22.78) | 526.00 (22.61) | 88.00 (23.85) |  |
| Wrist pain, n (%) | |  |  | 0.588 |
| No | 2,218.00 (82.30) | 1,918.00 (82.46) | 300.00 (81.30) | |
| Yes | 477.00 (17.70) | 408.00 (17.54) | 69.00 (18.70) |  |
| Finger pain, n (%) | |  |  | 0.078 |
| No | 2,186.00 (81.11) | 1,899.00 (81.64) | 287.00 (77.78) | |
| Yes | 509.00 (18.89) | 427.00 (18.36) | 82.00 (22.22) |  |
| Back pain, n (%) | |  |  | 0.081 |
| No | 2,082.00 (77.25) | 1,810.00 (77.82) | 272.00 (73.71) | |
| Yes | 613.00 (22.75) | 516.00 (22.18) | 97.00 (26.29) |  |
| Lumbar pain, n (%) | |  |  | 0.033 |
| No | 1,676.00 (62.19) | 1,465.00 (62.98) | 211.00 (57.18) | |
| Yes | 1,019.00 (37.81) | 861.00 (37.02) | 158.00 (42.82) | |
| Hip pain, n (%) | |  |  | 0.034 |
| No | 2,349.00 (87.16) | 2,040.00 (87.70) | 309.00 (83.74) | |
| Yes | 346.00 (12.84) | 286.00 (12.30) | 60.00 (16.26) |  |
| Leg pain, n (%) | |  |  | 0.003 |
| No | 1,852.00 (68.72) | 1,623.00 (69.78) | 229.00 (62.06) | |
| Yes | 843.00 (31.28) | 703.00 (30.22) | 140.00 (37.94) | |
| Knee pain, n (%) | |  |  | 0.016 |
| No | 1,777.00 (65.94) | 1,554.00 (66.81) | 223.00 (60.43) | |
| Yes | 918.00 (34.06) | 772.00 (33.19) | 146.00 (39.57) | |
| Ankle pain, n (%) | |  |  | 0.016 |
| No | 2,236.00 (82.97) | 1,946.00 (83.66) | 290.00 (78.59) | |
| Yes | 459.00 (17.03) | 380.00 (16.34) | 79.00 (21.41) |  |
| Grip strength, kg, median (IQR) | 27.80 (22.20,34.00) | 28.00 (22.80,34.50) | 25.00 (20.00,31.00) | <0.001 |
| TyG index, mean (SD) | 8.73 (0.62) | 8.72 (0.62) | 8.81 (0.62) | 0.010 |
| TyG index class, n (%) | |  |  | 0.035 |
| Q1 | 674.00 (25.01) | 602.00 (25.88) | 72.00 (19.51) |  |
| Q2 | 674.00 (25.01) | 582.00 (25.02) | 92.00 (24.93) |  |
| Q3 | 674.00 (25.01) | 577.00 (24.81) | 97.00 (26.29) |  |
| Q4 | 673.00 (24.97) | 565.00 (24.29) | 108.00 (29.27) | |
| TyG, Triglyceride-Glucose Index; ADL, Activities of Daily Living; BMI, Body mass index; Q, Quartiles; IQR, Interquartile Range; SD, standard deviation. | | | | |

| Supplementary Table 2 Sensitivity analysis of the association between TyG index and ADL disability | | | | | | |
| --- | --- | --- | --- | --- | --- | --- |
|  | Continuous variable | TyG index (HR, 95% CI) | | | | *P* for trend^a^ |
|  |  | Low TyG | Moderate-Low TyG | Moderate-High TyG | High TyG |  |
| Model 1 HR(95%CI) | 1.43 (1.10, 1.86)^**^ | Ref. | 1.33 (0.82, 2.15) | 1.74 (1.09, 2.78)^*^ | 2.10 (1.29, 3.42)^**^ | 0.001 |
| Model 2 HR(95%CI) | 1.46 (1.22, 1.74)^***^ | Ref. | 1.57 (1.13, 2.18)^**^ | 1.91 (1.38, 2.67)^***^ | 2.29 (1.62, 3.24)^***^ | <0.001 |
| Model 3 HR(95%CI) | 1.24 (1.04, 1.49)^*^ | Ref. | 1.28 (0.93, 1.74) | 1.38 (1.00, 1.89)^*^ | 1.60 (1.15, 2.22)^**^ | 0.005 |
| Model 4 HR(95%CI) | 1.18 (1.01, 1.42)^*^ | Ref. | 1.24 (0.90, 1.68) | 1.32 (1.01, 1.77)^*^ | 1.53 (1.11, 2.13)^**^ | 0.006 |
| Low TyG: 7.29<TyG≤8.28, Moderate-Low TyG: 8.28<TyG≤8.65, Moderate-High TyG: 8.65<TyG≤9.11, High TyG: 9.11<TyG≤11.23  TyG, Triglyceride-Glucose Index; ADL, Activities of Daily Living; CI, confidence interval; HR, Hazard ratio. Ref, Reference  Model 1: The Cox model was fitted using unimputed raw data to assess potential bias introduced by missing values (Outliers were treated as missing values).  Model 2: A robust weighted Cox proportional hazards model was employed, with weights derived from deviance residuals, to mitigate the influence of outliers on effect estimates.  Model 3: Covariates were adjusted based on the minimal sufficient adjustment set (MSAS) identified via causal directed acyclic graphs (DAGs), ensuring robust causal inference.  Model 4: The Fine-Gray subdistribution hazards model was applied to analyze the risk of the ADL disability, treating death as a competing event.  Models 1, 2, and 4 were adjusted for age, sex(female/male), Residence(urban/rural), education(illiteracy/elementary school/middle school/high school or above), Income,bmi, smoking(no/yes), drinking(no/yes), hypertension(no/yes), diabetes(no/yes), headache (no/yes), shoulder pain (no/yes), arm pain (no/yes), wrist pain (no/yes), finger pain (no/yes), back pain (no/yes), lumbar pain (no/yes), hip pain (no/yes), leg pain (no/yes), knee pain (no/yes), ankle pain (no/yes) and grip strength.  Model 3 (DAGs-based MSAS adjustment) was adjusted for age, sex(female/male), education(illiteracy/elementary school/middle school/high school or above), Income,bmi, diabetes(no/yes), finger pain (no/yes) and knee pain (no/yes).  ^a^ *P* for trend was estimated by including the quartile of the TyG index as a continuous variable  ^*^*P*<0.05,^**^*P*<0.01,^***^*P*<0.001 | | | | | | |

# Supplementary Figure


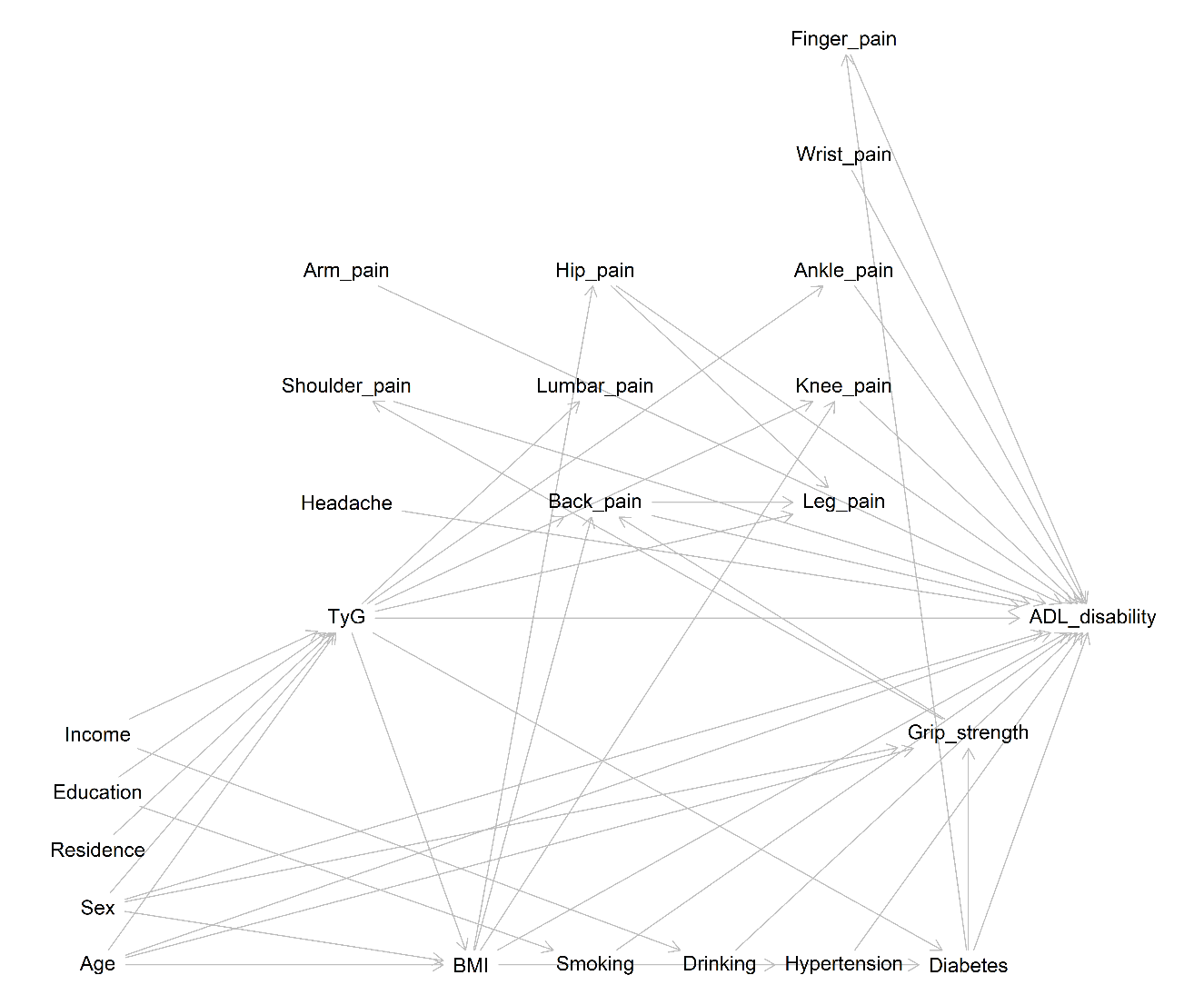


Supplementary Figure 1. Causal directed acyclic graph of the association between TyG index and ADL disability.

TyG, Triglyceride-Glucose Index; ADL, Activities of Daily Living; BMI, Body mass index
